# Multi‐networks connectivity at baseline predicts the clinical efficacy of left angular gyrus‐navigated rTMS in the spectrum of Alzheimer's disease: A sham‐controlled study

**DOI:** 10.1111/cns.14177

**Published:** 2023-03-21

**Authors:** Hai‐Feng Chen, Xiao‐Ning Sheng, Zhi‐Yuan Yang, Peng‐Fei Shao, Heng‐Heng Xu, Ruo‐Meng Qin, Hui Zhao, Feng Bai

**Affiliations:** ^1^ Department of Neurology, Drum Tower Hospital, Medical School and The State Key Laboratory of Pharmaceutical Biotechnology, Institute of Brain Science Nanjing University Nanjing China; ^2^ Nanjing Drum Tower Hospital Clinical College of Traditional Chinese and Western Medicine Nanjing University of Chinese Medicine Nanjing China; ^3^ Jiangsu Key Laboratory of Molecular Medicine Medical School of Nanjing University Nanjing China; ^4^ Jiangsu Province Stroke Center for Diagnosis and Therapy Nanjing China; ^5^ Nanjing Neuropsychiatry Clinic Medical Center Nanjing China; ^6^ Geriatric Medicine Center, Affiliated Taikang Xianlin Drum Tower Hospital Medical School of Nanjing University Nanjing China

**Keywords:** Alzheimer's disease, connectome‐based predictive modeling, default mode network, dorsal attention network, repetitive transcranial magnetic stimulation

## Abstract

**Introduction:**

Neuro‐navigated repetitive transcranial magnetic stimulation (rTMS) is effective in alleviating cognitive deficits in Alzheimer's disease (AD). However, the strategy for target determination and the mechanisms for cognitive improvement remain unclear.

**Methods:**

One hundred and thirteen elderly subjects were recruited in this study, including both cross‐sectional (*n* = 79) and longitudinal experiments (the rTMS group: *n* = 24; the sham group: *n* = 10). The cross‐sectional experiment explored the precise intervention target based on the cortical–hippocampal network. The longitudinal experiment investigated the clinical efficacy of neuro‐navigated rTMS treatment over a four‐week period and explored its underlying neural mechanism using seed‐based and network‐based analysis. Finally, we applied connectome‐based predictive modeling to predict the rTMS response using these functional features at baseline.

**Results:**

RTMS at a targeted site of the left angular gyrus (MNI: −45, −67, 38) significantly induced cognitive improvement in memory and language function (*p* < 0.001). The improved cognition correlated with the default mode network (DMN) subsystems. Furthermore, the connectivity patterns of DMN subsystems (*r* = 0.52, *p* = 0.01) or large‐scale networks (*r* = 0.85, *p* = 0.001) at baseline significantly predicted the Δ language cognition after the rTMS treatment. The connectivity patterns of DMN subsystems (*r* = 0.47, *p* = 0.019) or large‐scale networks (*r* = 0.80, *p* = 0.001) at baseline could predict the Δ memory cognition after the rTMS treatment.

**Conclusion:**

These findings suggest that neuro‐navigated rTMS targeting the left angular gyrus could improve cognitive function in AD patients. Importantly, dynamic regulation of the intra‐ and inter‐DMN at baseline may represent a potential predictor for favorable rTMS treatment response in patients with cognitive impairment.

## Graphical abstract

A summary of the study design and participant flow through the study. (A) Flow chat of enrollment and treatment in the longitudinal experiment; (B) and (C) seed‐based analysis and network‐based analysis; (D) predicting the therapeutic effect of rTMS; Abbreviations: LOO, leave‐one‐out; CPM, connectome‐based predictive modeling; rTMS, repetitive transcranial magnetic stimulation.

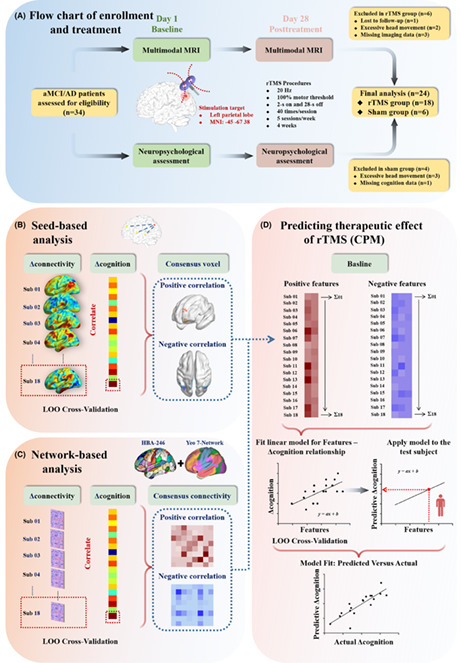

## 1 INTRODUCTION

Alzheimer's disease (AD) is a progressive neurodegenerative disorder worldwide. According to the Alzheimer's Association estimates, there were 5.8 million Americans after the age of 65 living with AD in 2020.^1^ The latest epidemiological study reported that the prevalence of AD and mild cognitive impairment (MCI) are estimated to be 3.9% (9.83 million) and 15.5% (38.77 million), respectively, among adults aged 60 and older in China.^2^ Most current therapies for AD are primarily concentrated on delaying cognitive deficits by medications, but most of them have failed.^3^ The narrow selectivity of AD treatment has aroused an increased interest in nonpharmacological treatment strategies. Repetitive transcranial magnetic stimulation (rTMS), the most commonly applied noninvasive brain stimulation technology, has gained increasing attention as a promising tool for AD treatment. Previous studies have shown that rTMS may improve cognitive performance in the AD continuum.^4^ However, in‐depth research on the potential mechanisms of rTMS interventions has been rare, and there is an imperative need to identify biomarkers for predicting treatment efficacy to guide individual therapy.

Repetitive transcranial magnetic stimulation has been reported as a noninvasive and safe approach in which the magnetic field penetrates the scalp and generates electrical current in the specific target cortex to modulate and balance the activity of cortical neurons.^5^, ^6^ It should be noted that the potential effects of rTMS spread from the stimulation site to anatomically distant connected areas, providing an opportunity to apply rTMS at one site in a neural network, also called “entry ports,” to regulate plasticity in specific functional networks along cortico‐cortical connections.^7^ Cortical plasticity refers to the ability of the brain to modify its structure and function through synaptic connections, which plays a pivotal role in sustaining complex cognitive functions.^8^ Recently, two pairwise meta‐analyses of randomized controlled trials demonstrated that application of rTMS conveys positive effects in cognitive rehabilitation in the spectrum of AD.^9^, ^10^ Improvements not only in general cognitive function but also in specific cognitive domains (e.g., memory function, information processing speed, language function) have been demonstrated in previous researches.^11^, ^12^, ^13^, ^14^ Despite the potential therapeutic effect of rTMS to modify neuroplasticity, the neural mechanisms underlying cognitive enhancement remain largely unknown. Resting‐state functional magnetic resonance imaging (rs‐fMRI) analysis has enabled us to further understand the underlying mechanisms of functional cortical reorganization during rTMS therapy.

To date, most of current researches on rTMS intervention of AD have chosen the left dorsolateral prefrontal cortex (DLPFC) as the stimulation site due to its cortical plasticity and important role in cognitive function.^15^, ^16^, ^17^ The DLPFC is a cortical hub within the frontoparietal network (FPN). From the early stages of AD, pathological abnormalities affect the posterior cingulate gyrus, medial prefrontal region, precuneus, medial temporal lobe (MTL), and lateral posterior parietal cortex.^18^ These alterations are paralleled by an initial impairment of intrinsic functional connectivity, as revealed by changes in the default mode network (DMN). For patients with AD, abnormal connectivity referring to the DMN may represent a treatment site for rTMS.^19^, ^20^ Brain regions that are accessible include the angular cortex, which in turn exhibit connectivity to other DMN subregions. Stimulation of the left angular cortex gained proof of concept in an rTMS‐fMRI study, and a multiple rTMS session protocol over the left angular cortex (based on connectivity in the cortical–hippocampal network) led to significant enhancement in memory function.^19^ Thus, the left angular cortex related to the DMN could be an appropriate potential site and an intermediate imaging marker for interventions aimed at improving memory function in AD. Furthermore, the exact patterns of functional rewiring of the brain connectome (DMN and other networks) underlying the neural mechanism of cognitive improvement need to be investigated.

In this research, our aims were first to investigate the clinical efficacy of the neuro‐navigated rTMS protocol to improve cognition in AD patients at the target located in the left angular cortex. Secondly, we aimed to explore the potential mechanisms using fMRI analysis. Finally, we aimed to predict the therapeutic efficacy of rTMS interventions using baseline functional connectivity. We hypothesized that changes in DMN‐related connectivity are involved in the potential mechanisms of rTMS and would help identify who responds preferentially to rTMS through assessment of baseline functional connectivity.

## 2 MATERIALS AND METHODS

### 2.1 Experimental design

**FIGURE 1:**
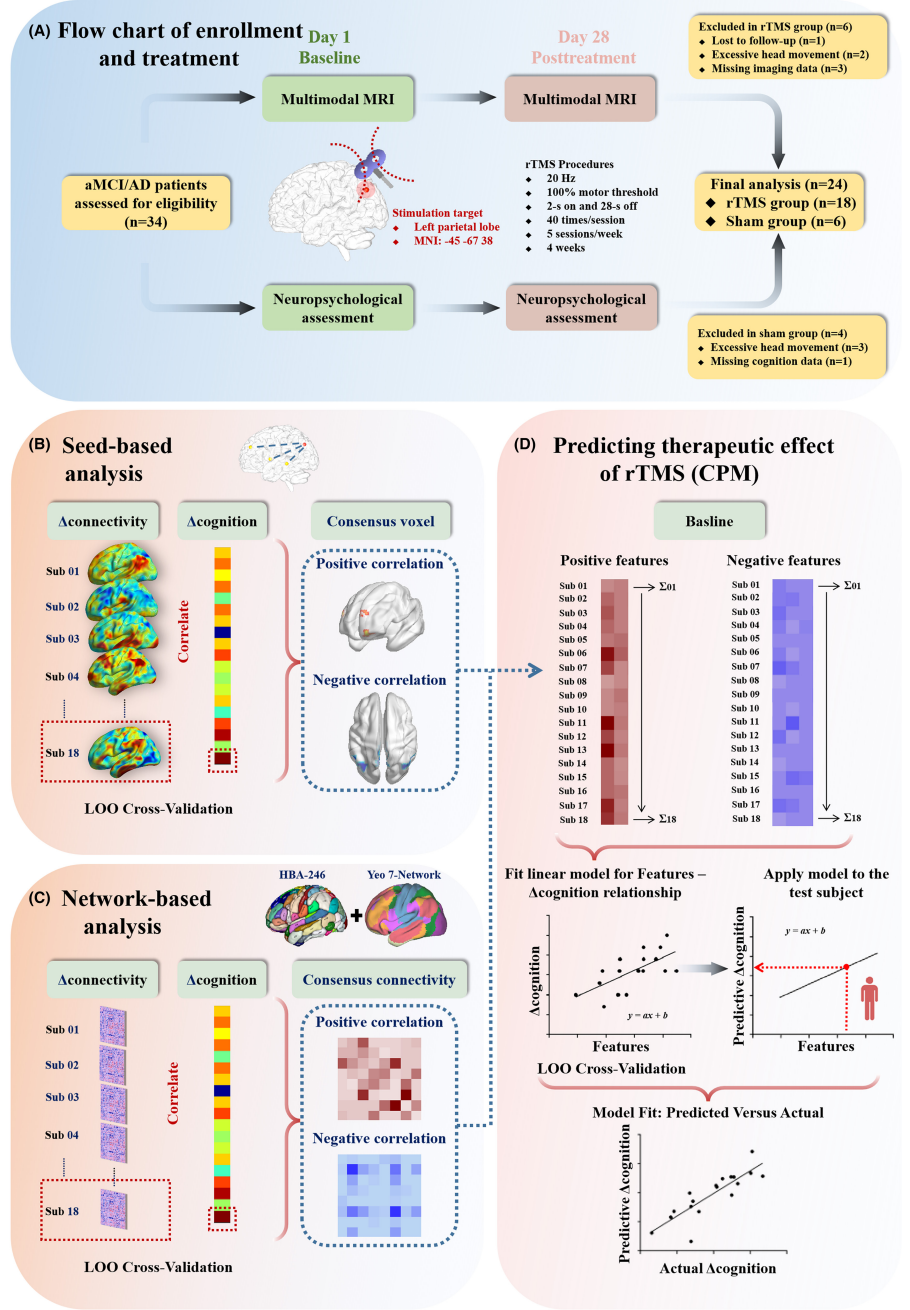
Summary of the study design and participant flow through the study. (A) Flow chat of enrollment and treatment in the longitudinal experiment; (B) and (C) seed‐based analysis and network‐based analysis; (D) predicting the therapeutic effect of rTMS. CPM, connectome‐based predictive modeling; LOO, leave‐one‐out; rTMS, repetitive transcranial magnetic stimulation.

This study included both cross‐sectional and longitudinal parts. The cross‐sectional part investigated the stimulation location, and the longitudinal part explored the efficacy of rTMS treatment and its underlying mechanism using rs‐fMRI techniques (Figure 1A–C). Finally, the connectome‐based predictive modeling (CPM) was used to predict the rTMS treatment effect using functional connectivity at baseline (Figure 1D). This study was approved by the ethics committee of Nanjing Drum Tower Hospital. All subjects gave written informed consent according to the Declaration of Helsinki.

### 2.2 Participants

One hundred and thirteen subjects participated in this research. The cross‐sectional experiment included 26 HC, 30 aMCI, and 23 AD subjects, and the longitudinal experiment included the rTMS group (15 aMCI and 9 AD subjects) and the sham group (8 aMCI and 2 AD subjects). The participants in cross‐sectional experiment were recruited from the Nanjing Drum Tower Hospital between January 2017 and December 2018. The participants in longitudinal experiment (e.g., rTMS experiment) were recruited between January 2019 and March 2022. The inclusion criteria of HC, aMCI and AD is described in the Appendix S1.^21^, ^22^, ^23^ Exclusion criteria included other neurological diseases, treatment with a cognitive enhancer (i.e., donepezil hydrochloride or memantine hydrochloride) up to 2 weeks before rTMS stimulation, or any contraindication for MRI or rTMS.

### 2.3 Neuropsychological measurement

In this study, general cognitive performance was evaluated using the clinical dementia rating (CDR), the Beijing version of the Montreal Cognitive Assessment (MoCA‐BJ) and mini mental state examination (MMSE). Multiple cognition domains were also evaluated, and the detailed information is described in the Appendix S1. All measurements were practiced once prior to study commencement to reduce practice effects that were most prominent between the first and second tests.

### 2.4 MRI scanning

The multimodal MRI data were acquired using a Philips Medical Systems 3.0T scanner. In the longitudinal experiment, multimodal MRI scans were acquired before and after rTMS treatment. The protocol included a gradient‐recalled echo planar imaging and a high‐resolution 3D T1‐weighted imaging sequence. Detailed sequence parameters are provided in the Appendix S1.

### 2.5 MRI data preprocessing

The rs‐fMRI data preprocessing was conducted using the DPABI software (V4.3, http://rfmri.org/dpabi/), implemented in MATLAB. The main preprocessing steps included removing the first 10 volumes, slice‐timing correction, head‐motion correction, normalization to the Montreal Neurological Institute (MNI) space (a resolution of 3 mm isotropic voxels), spatial smoothing (6 × 6 × 6 mm^3^), band‐pass filtering (0.01–0.1 Hz), and multiple linear regression analysis, including regressing out the Friston 24 parameters, cerebrospinal fluid, and white matter signals. Individuals who performed a displacement >2 mm or an angular rotation >2° in any direction with mean frame‐wise displacement proposed by Jenkinson (FD‐Jenkinson) > 0.25 were excluded.^24^, ^25^

### 2.6 Identification of stimulation locations in the cross‐sectional experiment

We identified the stimulation location according to the hippocampal resting‐state functional connectivity obtained in the cross‐sectional experiment. Seventy‐nine elderly subjects were recruited in the cross‐sectional experiment, including 26 HC, 30 aMCI, and 23 AD subjects. Demographic and clinical data in this cross‐sectional experiment are summarized in Table S1. For each subject, fMRI data were used to generate seed‐based connectivity maps using the left hippocampus as the seed (as shown in Anatomical Automatic Labelling 90). Firstly, we determined the stimulation location in the lateral parietal cortex because it is the portion of the hippocampal intrinsic fMRI connectivity networks that can be best targeted with rTMS because it is superficial. The only other moderately superficial region that is robustly within the hippocampal fMRI network is the medial prefrontal cortex, and we avoided stimulation of this location because the cortical surface is oriented perpendicular to the stimulating coil; therefore, rTMS might be minimally effective for this region. Secondly, the left brain hemisphere was selected because of the known role of the left parietal cortex in memory function; therefore, hippocampal connectivity with the lateral parietal cortex is primarily ipsilateral. The detailed processing was described as follows.

**FIGURE 2:**
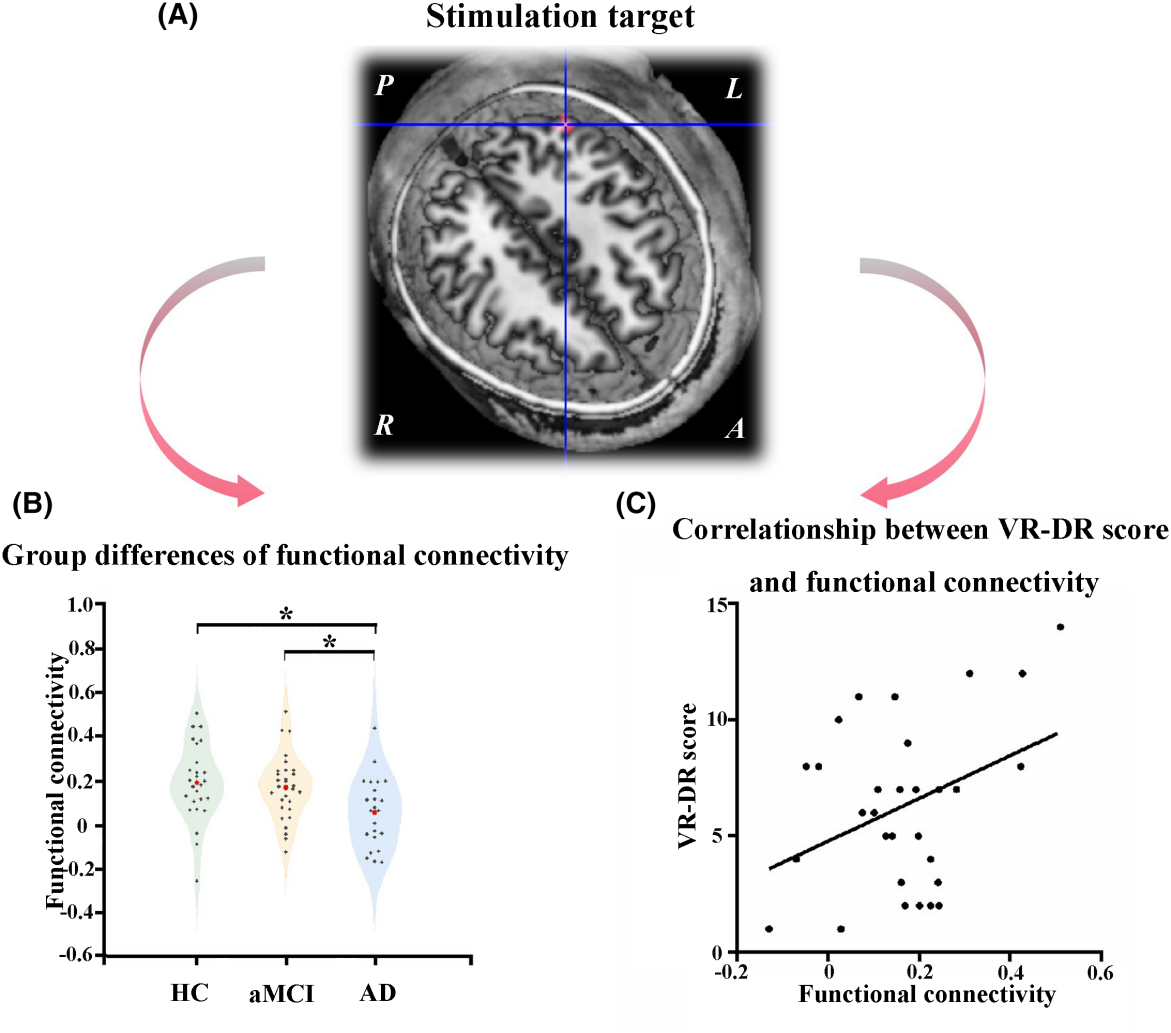
Cross‐sectional experiment—identification of stimulation locations. (A) One cluster showed significant differences among HC, aMCI, and AD participants (*p* < 0.05, cluster size >20 voxels, 594 mm^3^). (B) The post hoc test indicated AD and aMCI showed the gradually decreased functional connectivity between left hippocampus and left parietal. (C) The alteration of the left parietal with the left hippocampus was positively correlated with the changes in VR‐DR scores in aMCI subjects (*r* = 0.374, *p* = 0.042). AD, Alzheimer's disease; aMCI, amnestic mild cognitive impairment; HC, healthy controls; VR‐DR, visual reproduction‐delayed recall.

Seed‐based functional connectivity analysis was conducted using REST software (http://www.restfmri.net/forum/REST_V1.8). The left hippocampus (as shown in Anatomical Automatic Labelling 90) was used as a seed region for the functional connectivity analysis. With gender, age, and years of education as covariates, one‐way ANCOVA analysis was performed in this study. No significant difference in FD‐Jenkinson among HC (0.14 ± 0.06), aMCI (0.13 ± 0.07), and AD (0.15 ± 0.06) (F = 0.772, *p* = 0.465). Therefore, we did not add head motion (e.g., FD‐Jenkinson) as covariates. We found that only one cluster exhibited group differences among HC, aMCI, and AD participants (*p* < 0.05, cluster size = 594 mm^3^) (Figure 2A). The post hoc test revealed that AD presented lower functional connectivity between the left hippocampus and this cluster compared to HC and aMCI individuals (Figure 2B). Alteration of the left hippocampus with this cluster was positively related to changes in VR‐DR scores in aMCI subjects (*r* = 0.374, *p* = 0.042; Figure 2C). Thus, the stimulation target was defined as a sphere of 6‐mm radius centered at the MNI coordinate [−45, −67, 38], which was the center of this cluster located in the angular cortex belonging to the parietal region. We also performed ANCOVA analysis with gender, age, years of education, and head motion (e.g., FD‐Jenkinson) as covariates. This significantly different cluster was stable, and the result was described in the Appendix S1 (this cluster as shown in Figure S4).

### 2.7 Neuro‐navigated rTMS

Repetitive transcranial magnetic stimulation was delivered using YIRUIDE CCY‐IV model stimulator. For rTMS, the motor threshold was tested in all participants from the longitudinal experiment over the left motor cortex by identifying the lowest intensity that induced a motor response in the right abductor pollicis brevis muscle, which produced five motor‐evoked responses of at least 50 μV in 10 consecutive trials. The specific rTMS parameters of this study were 20 Hz stimulation frequency, 2 s stimulation, and 28 s inter‐interval. Stimulation intensity was determined in 100% of resting motor threshold with 20 min stimulation duration and a total of 1600 pulses for one session per day, 5 days per week for a 4‐week treatment course (5 days per week, from Monday to Friday, with 2 days off each weekend).^19^, ^26^, ^27^ All participants received the same rTMS treatment parameters in the morning.

The stimulation region is defined at MNI coordinates [−45, −67, 38], based on the result of cross‐sectional experiments. This coordinate was transformed into each individual's 3DT1 space by applying an inverse matrix in the TMStarget software and the SPM12 software (http://www.fil.ion.ucl.ac.uk/spm/software/spm12/). Then, each participant's stimulation target was imported to a neuro‐navigation system (Visor 2.0, Enschede, the Netherlands). The rTMS treatment procession was in real time to ensure accuracy of the stimulation. In the sham group, stimulation was applied using the same rTMS parameters with the sham TMS coil positioned to the same target area but did not induce treatment effect.

### 2.8 Seed‐based analysis in the longitudinal experiment

A spherical region of interest (ROI, radius = 6 mm) as a seed was centered at the stimulation target [−45, −67, 38] located in the left angular cortex. Pearson correlation analysis was conducted between the time series in the seed region and other whole‐brain voxels. Finally, to improve the normality of the functional connectivity, Fisher's z‐transformation was performed.

### 2.9 Network‐based analysis in the longitudinal experiment

The Human Brainnetome Atlas (https://atlas.brainnetome.org/) was used to divide the entire brain into 246 ROIs. The average BOLD time series of 246 nodes within the 7‐network functional atlas of Yeo et al.^28^ and subcortical network (SN) were extracted. The eight canonical neural networks included the SN, limbic network (LN), visual network (VN), FPN, DMN, somatomotor network (SMN), dorsal attention network (DAN), and salience/ventral attention network (SVAN). To define the network edge, we calculated the Pearson correlation coefficient of the average time series between each pair of 246 ROIs. Only those edges whose corresponding *p* were lower than a statistical threshold (*p* < 0.05, false discovery rate [FDR]‐corrected) were retained.^24^

### 2.10 Statistical analysis

Statistical analyses of demographic characteristics and cognitive performance were performed using SPSS software (Version 22, IBM Corp., USA). The Shapiro–Wilk test was used to assess whether the data followed a normal/Gaussian distribution. Differences in cognitive performance and head motion before and after rTMS treatment were analyzed by paired *t*‐tests.

Regression analysis was performed to evaluate the relation between changes in seed‐based and network‐based functional connectivity and cognitive performance after rTMS treatment (Figure 1B,C). Due to the limited number of samples in this study, a leave‐one‐out (LOO) cross‐validation strategy was used to investigate consensus connectivity. First, we suppose there are *N* samples in this study. In each LOO cross‐validation, one sample is left out and the remaining *N*–1 samples are used to conduct the correlation analysis. Finally, the correlation analysis was performed *N* times. The consensus connectivity, including positive and negative networks, refers to the connections that could be identified in each LOO cross‐validation.

### 2.11 Individualized prediction of rTMS treatment effects

In this study, CPM was used to estimate rTMS treatment effects using either seed‐based or network‐based connectivity at baseline (Figure 1D).^29^ Briefly, CPM takes cognitive performance and group connectivity matrices as input to build a predictive model of the cognition from functional connectivity matrices. The regression analyses were used to identify positive and negative predictive networks between connectivity matrix and cognitive data from the training dataset (*p* < 0.05). In this study, we used positive and negative networks obtained from seed‐based and network‐based analyses as masks of predictive networks. Then, we extracted the predictive networks based on this mask at baseline. Cognitive data represent changes in cognitive performance after rTMS treatment. Single‐subject summary statistics were created as the sum of the significant edge weights in each network and were entered into predictive models that assumed linear relationships with cognitive data. The resultant polynomial coefficients were then applied to the testing data to predict the cognitive score. Based on LOO cross‐validation strategy, a single individual's predicted value is obtained by taking the data from all other individuals as the training data until all individuals have a predicted value in an iterative manner. The prediction performance was assessed by calculating Pearson correlation coefficients between predicted and observed cognitive data.

Analyses in the LOO cross‐validation are not wholly independent and the number of degrees of freedom is thus overestimated for parametric *p*‐values based on correlation. Therefore, we performed permutation testing. We randomly shuffled the correspondence between cognitive performance and functional connectivity matrices 1000 times and reran the CPM analysis using the shuffled data to generate null distributions for significance testing.^30^ The *p* for LOO predictions were computed based on the null distributions.

## 3 RESULTS

### 3.1 Demographic and clinical characteristics

In the longitudinal experiment, six participants (2 aMCI and 4 AD patients) in the real treatment group were excluded from analysis because of lost to follow‐up (*n* = 1), excessive head‐motion artifacts (*n* = 2) and missing imaging data (*n* = 3). Four participants (2 aMCI and 2 AD patients) in the sham group were excluded from analysis because of excessive head‐motion artifacts (*n* = 3) and missing cognition data (*n* = 1). Thus, 18 participants (13 aMCI and 5 AD patients) in rTMS group and six participants (6 aMCI patients) in sham group were included in the final analysis. To avoid the confusion, we list the MMSE scores of aMCI and AD patients separately in the longitudinal experiment (shown in the Table S2).

Demographic and clinical data before and after stimulation in rTMS group and sham group are shown in Table 1. Due to AD and aMCI belonging to AD spectrum disorder and the limited sample size in the longitudinal experiment, we counted the demographic and clinical data of AD and aMCI together in Table 1. No significant difference from pre‐ to post‐treatment was found in the head motion parameter (i.e., mean frame‐wise displacement proposed by Jenkinson) (rTMS group: *t* = −0.44, *p* = 0.66; sham group: *t* = −0.48, *p* = 0.65). Significant improvements of cognitive performance were seen after post‐stimulation in rTMS group, using a paired *t*‐test. In detail, we found that the stimulation target induced cognitive improvement in the MoCA‐BJ (*t* = 4.64, *p* < 0.001), the memory function (*t* = 5.36, *p* < 0.001), and the language function (*t* = 4.74, *p* < 0.001). We observed no significant changes in the MMSE (*t* = 1.73, *p* = 0.10), visuospatial function (*t* = 1.11, *p* = 0.28), information processing speed (*t* = 1.30, *p* = 0.21), and executive function (*t* = 1.99, *p* = 0.06) after the rTMS stimulation (Figure S1). In contrast, there was no significant difference in post‐treatment compared with baseline in the sham group (Table 1). In addition, we re‐analyzed aMCI and AD data separately in the rTMS treatment group. We found that the AD group (*n* = 13) showed cognitive improvement in the MoCA‐BJ (*t* = 3.32, *p* = 0.006), the memory function (*t* = 4.91, *p* < 0.001), and the language function (*t* = 6.01, *p* < 0.001) after rTMS treatment. In the aMCI group (*n* = 5), the MoCA‐BJ (*t* = 3.65, *p* = 0.02), the memory function (*t* = 3.03, *p* = 0.04), and the Boston Naming Test of language function (*t* = 2.81, *p* = 0.04) were also significantly improved after rTMS treatment. However, aMCI may specifically get better beneficial effect than AD after the rTMS treatment. The detailed demographic and cognitive data of AD and aMCI are summarized in Table S3. Demographic and clinical characteristics in the cross‐sectional experiment are summarized in Appendix S1.

**TABLE 1:** Demographic and neuropsychological data in the longitudinal experiment.

Items	rTMS (aMCI = 13, AD = 5)		*t/p*	Sham (aMCI = 6)		*t/p*
	Pre	Post		Pre	Post	
Demographics						
Age (years)	66.67 ± 7.48		–	67.17 ± 8.75		–
Education (years)	10.97 ± 4.08		–	11.33 ± 3.62		–
Gender (male/female)	7/11		–	4/2		–
Head motion						
Mean FD_Jenkinson	0.12 ± 0.05	0.11 ± 0.05	−0.44/0.66	0.14 ± 0.07	0.15 ± 0.07	−0.48/0.65
General cognition						
MMSE (raw score)	25.06 ± 4.95	26.06 ± 4.37	1.73/0.10	29.33 ± 1.03	28.83 ± 1.17	2.24/0.08
MoCA‐BJ (raw score)	20.11 ± 5.87	23.17 ± 5.01	4.64/<0.001*	24.83 ± 3.66	25.67 ± 3.78	−0.58/0.59
Multiple cognitive domain						
Memory Function (z‐score)	−0.24 ± 0.78	0.24 ± 0.88	5.36/<0.001*	−0.25 ± 0.43	0.25 ± 0.83	−1.54/0.18
AVLT‐DR (raw score)	2.44 ± 2.81	4.78 ± 3.46	4.68/<0.001*	5.33 ± 1.37	7.00 ± 2.61	−2.08/0.09
AVLT‐R (raw score)	15.44 ± 6.27	16.83 ± 5.83	1.81/0.09	20.50 ± 2.43	20.17 ± 2.04	0.26/0.81
VR‐DR (raw score)	3.94 ± 4.05	6.17 ± 4.73	2.86/0.01*	6.00 ± 3.16	9.50 ± 4.09	−3.31/0.02*
Visuospatial Function (*z*‐score)	−0.07 ± 1.06	0.07 ± 0.84	1.11/0.28	−0.27 ± 1.25	0.27 ± 0.38	−0.94/0.39
CDT (raw score)	3.22 ± 1.26	3.44 ± 0.86	0.89/0.39	3.33 ± 0.82	3.67 ± 0.52	−0.79/0.47
VR‐C (raw score)	12.78 ± 2.71	13.00 ± 2.70	1.29/0.22	13.67 ± 0.82	14.00 ± 0.00	−1.00/0.36
Information Processing Speed (*z*‐score)	−0.10 ± 0.81	0.10 ± 0.96	1.30/0.21	−0.07 ± 0.97	0.07 ± 0.97	−0.76/0.48
TMT‐A (raw score)	97.17 ± 78.05	94.00 ± 68.44	−0.37/0.71	71.33 ± 56.49	61.50 ± 32.36	0.49/0.64
Stroop A (raw score)	27.94 ± 15.46	24.50 ± 11.66	−1.32/0.20	25.00 ± 10.83	23.00 ± 6.75	0.66/0.54
Stroop B (raw score)	43.17 ± 57.13	33.39 ± 28.33	−1.37/0.19	25.33 ± 9.16	22.82 ± 4.67	1.02/0.36
Language Function (*z*‐score)	−0.24 ± 0.73	0.24 ± 0.77	4.74/<0.001*	−0.17 ± 0.89	0.17 ± 0.93	−2.62/0.05
CVF (raw score)	14.56 ± 4.61	16.33 ± 4.65	2.18/0.04*	16.50 ± 4.85	20.50 ± 6.25	−2.62/0.05
BNT (raw score)	46.83 ± 8.40	51.33 ± 6.98	4.57/<0.001*	51.00 ± 9.42	51.00 ± 9.42	−−/−−
Executive Function (*z*‐score)	−0.12 ± 0.80	0.12 ± 0.70	1.99/0.06	−0.04 ± 0.66	0.04 ± 0.79	−0.27/0.80
TMT‐B (raw score)	208.83 ± 132.18	201.33 ± 154.49	−0.28/0.79	169.33 ± 138.47	115.67 ± 66.08	1.78/0.14
Stroop C (raw score)	51.33 ± 45.95	46.61 ± 29.75	−0.85/0.41	34.00 ± 10.81	29.67 ± 12.34	0.97/0.38
DST‐backward (raw score)	3.89 ± 1.37	4.44 ± 1.04	2.05/0.06	4.17 ± 0.75	3.83 ± 0.75	1.00/0.36

*Note*: Values are presented as the mean ± standard deviation (SD). The *p*‐value was obtained by paired *t*‐test. * indicates a statistical difference between baseline and post‐treatment, *p* < 0.05.

Abbreviations: AD, Alzheimer's disease; aMCI, amnestic mild cognitive impairment; AVLT‐DR, Auditory Verbal Learning Test‐delayed recall; AVLT‐R, Auditory Verbal Learning Test‐recognition; BNT, Boston Naming Test; CDT, Clock Drawing Test; CVF, category verbal fluency; DST, Digit Span Test; FD, framewise displacement; MMSE, mini mental state examination; MoCA‐BJ, Beijing version of the Montreal Cognitive Assessment; rTMS, repetitive transcranial magnetic stimulation; Stroop A, B and C, Stroop Color and Word Tests A, B, and C; TMT‐A and TMT‐B, Trail Making Test‐A and B; VR‐C, visual reproduction‐copy; VR‐DR, visual reproduction‐delay recall.

### 3.2 Seed‐based analysis and individualized prediction

**FIGURE 3:**
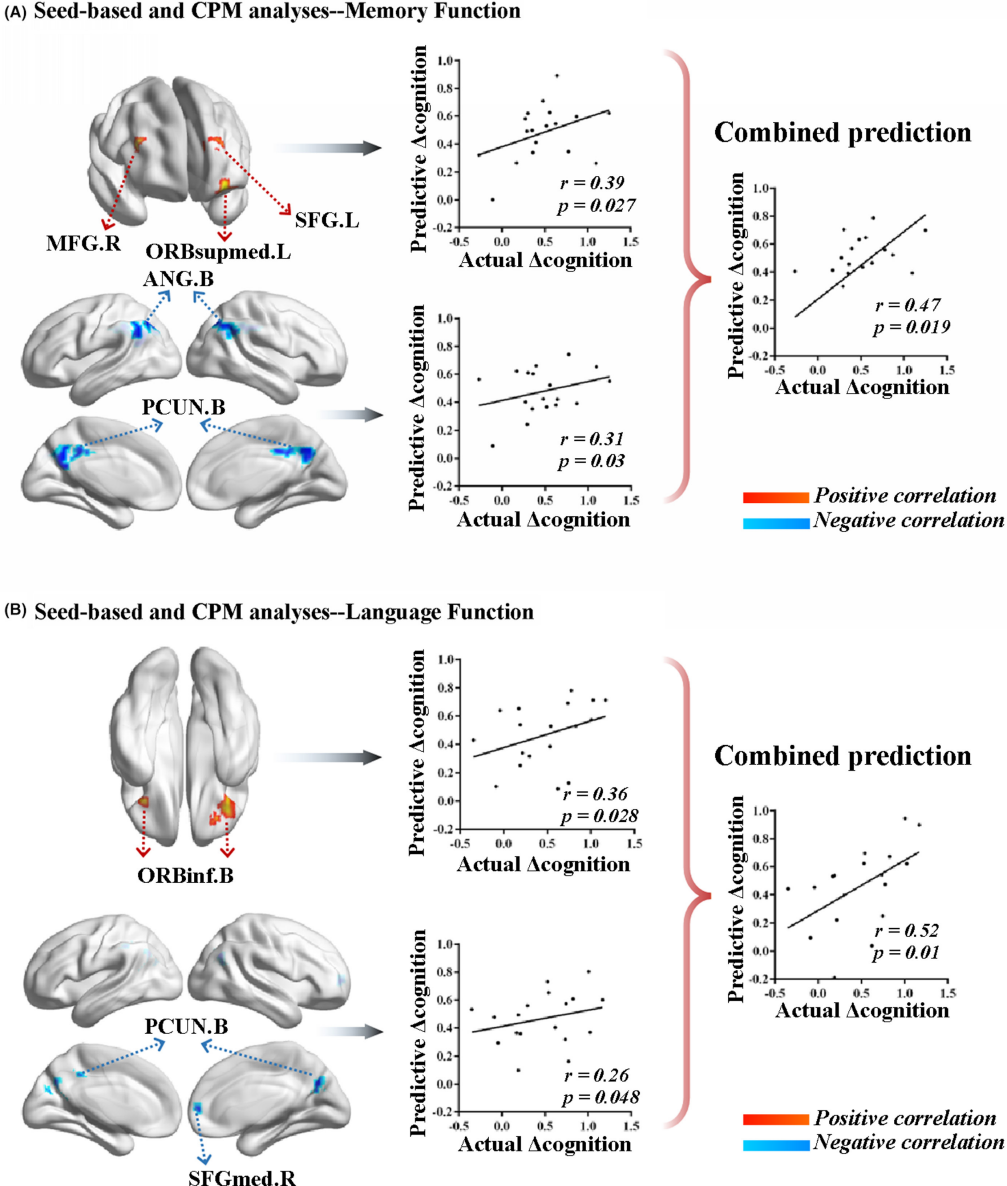
Results of seed‐based and CPM analysis. (A) Δ connectivity between the stimulation target and SFG.L, ORBsupmed.L, and MFG.R showed significantly positive correlation with Δ memory function (*p* < 0.05). Δ connectivity between the stimulation target and ANG.B and PCUN.B were negatively associated Δ memory function (*p* < 0.05). Predictive models obtained from above analyses significantly predicted Δ memory function after rTMS treatment based on seed‐based connectivity at baseline (positive network: *r* = 0.39, *p* = 0.027; negative network: *r* = 0.31, *p* = 0.03; combined networks: *r* = 0.47, *p* = 0.019). (B) Δ language cognition were positively correlated with Δ connectivity between the stimulation target and ORBinf.B, while negatively related to Δ connectivity between the stimulation target and PCUN.B and SFGmed.R (*p* < 0.05). The CPM method could predict Δ language cognition (positive network: *r* = 0.36, *p* = 0.028; negative network: *r* = 0.26, *p* = 0.048; combined networks: *r* = 0.52, *p* = 0.01). ANG.B, bilateral angular gyrus; CPM, connectome‐based predictive modeling; MFG.R, right middle frontal gyrus; ORBinf.B, bilateral orbital part of inferior frontal gyrus; ORBsupmed.L, left medial orbital part of superior frontal gyrus; PCUN.B, bilateral bilateral precuneus; SFG.L, left superior frontal gyrus; SFGmed.R, right medial part of superior frontal gyrus.

To explore the functional connectivity mechanism of rTMS treatment effects, regression analysis was used to examine the correlation between the changes in seed‐based functional connectivity and cognitive performance (i.e., between Δ connectivity and Δ cognition) after rTMS stimulation. First, we constructed functional networks based on the stimulation target before and after rTMS treatment (Figure S2). The stimulation‐based networks displayed a similar functional connectivity pattern with DMN. According to previous studies, the DMN were segregated into three distinct subsystems: the MTL subsystem, the core subsystem, and the dorsal medial prefrontal cortex (dMPFC) subsystem.^31^ Due to improvement of memory and language function after rTMS treatment, we then investigated the relationship between Δ connectivity (limited to the stimulation‐based network) and Δ cognition (Δ memory and Δ language cognition, respectively). According to LOO cross‐validation strategy, Δ connectivity between the stimulation target and the dMPFC subsystem [left superior frontal gyrus (SFG.L), left medial orbital part of SFG (ORBsupmed.L) and right middle frontal gyrus (MFG.R)] showed significantly positive correlation with Δ memory function (*p* < 0.05, Figure 3A). We found that Δ connectivity between the stimulation target and the core subsystem [bilateral angular gyrus (ANG.B) and bilateral bilateral precuneus (PCUN.B)] were negatively associated Δ memory function (*p* < 0.05, Figure 3A). In addition, Δ language cognition were positively related to Δ connectivity between the stimulation target and dMPFC subsystem [bilateral orbital part of inferior frontal gyrus (ORBinf.B)], while negatively related to Δ connectivity between the stimulation target and the core subsystem [PCUN.B and right medial part of superior frontal gyrus (SFGmed.R)] (*p* < 0.05, Figure 3B).

Next, the CPM method was applied to predict rTMS treatment effects. Predictive models obtained from above analyses significantly predicted Δ memory function after rTMS treatment based on seed‐based connectivity at baseline (positive network: *r* = 0.39, *p* = 0.027; negative network: *r* = 0.31, *p* = 0.03; combined networks: *r* = 0.47, *p* = 0.019; Figure 3A). In addition, the CPM method could successfully predict Δ language cognition (positive network: *r* = 0.36, *p* = 0.028; negative network: *r* = 0.26, *p* = 0.048; combined networks: *r* = 0.52, *p* = 0.01; Figure 3B).

### 3.3 Network‐based analysis and individualized prediction

**FIGURE 4:**
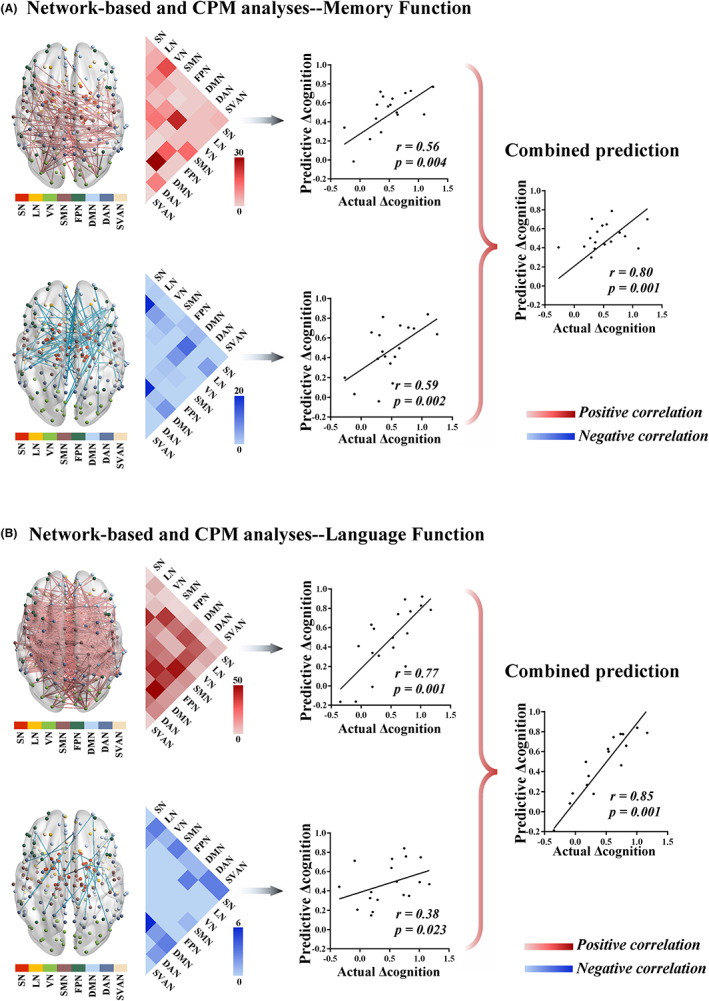
Results of network‐based and CPM analysis. (A) The spatial extent of both positive and negative networks correlated with Δ memory function included 218 edges (144 positive, 74 negative). The positive network included relatively more connections between DMN and DAN, and the negative network included relatively more within‐network connections for DMN and LN. The network‐based connectivity at baseline could predict Δ memory function after rTMS treatment (positive network: *r* = 0.56, *p* = 0.004; negative network: *r* = 0.59, *p* = 0.002; combined networks: *r* = 0.80, *p* = 0.001). (B) Δ language cognition were significantly related to 621 edges (591 positive, 30 negative). The positive network was further characterized by more connections between DMN and DAN, whereas the negative network included relatively more within‐network connections for DMN. The CPM method could significantly predict Δ language cognition (positive network: *r* = 0.77, *p* = 0.001; negative network: *r* = 0.38, *p* = 0.023; combined networks: *r* = 0.85, *p* = 0.001). CPM, connectome‐based predictive modeling; DAN, dorsal attention network; DMN, default mode network; LN, limbic network.

From the perspective of whole‐brain functional connectivity, we used regression analysis to explore the functional connectivity mechanism of rTMS treatment effects. First, we constructed whole‐brain functional networks before and after rTMS treatment (Figure S3). We then investigated the relationship between Δ connectivity and Δ cognition (Δ memory and Δ language cognition, respectively). The LOO cross‐validation method was applied to get the consensus connectivity. The spatial extent of both positive and negative networks correlated with Δ memory function included 218 edges (144 positive, 74 negative; Figure 4A). To facilitate characterization of rTMS treatment‐related networks, Figure 4A shows functional connectivity based on the number of connections within and between neural networks for the positive and negative networks correlated with Δ memory function. The positive network referred to relatively more connectivity between DMN and DAN, and the negative network referred to relatively more within‐network connectivity for DMN and LN (Figure 4A). In addition, Δ language cognition were significantly related to 621 edges (591 positive, 30 negative; Figure 4B). The positive network was further shown by more connectivity between DMN and DAN, whereas the negative network included relatively more within‐network connectivity for DMN (Figure 4B).

Next, the CPM method was used to predict rTMS treatment effects based on network‐based connectivity obtained from above analyses at baseline. The network‐based connectivity at baseline could predict Δ memory function after rTMS treatment (positive network: *r* = 0.56, *p* = 0.004; negative network: *r* = 0.59, *p* = 0.002; combined networks: *r* = 0.80, *p* = 0.001; Figure 4A). In addition, the CPM method could significantly predict Δ language cognition (positive network: *r* = 0.77, *p* = 0.001; negative network: *r* = 0.38, *p* = 0.023; combined networks: *r* = 0.85, *p* = 0.001; Figure 4B).

## 4 DISCUSSION

So far, there have been limited studies focusing on resting‐state network modifications caused by neuro‐navigated rTMS therapy in AD. Our findings indicated that rTMS intervention could improve cognition at the targeted location of the left angular cortex (MNI: −45, −67, 38). In addition, this study reveals a potential modulation mechanism of DMN subsystems and the large‐scale functional networks associated with the DMN in cognitive improvement. Moreover, dynamic regulation of the intra‐ and inter‐DMN at baseline may be assessed as a potential predictor of favorable rTMS treatment response in AD patients.

### 4.1 The left angular gyrus as a potential stimulation site

Alzheimer's disease is typically characterized by progressively impaired episodic memory retrieval and retention before involving more widespread cognitive domains.^32^ The influential notion showed that the hippocampus plays an important role in memory function by interacting with functionally distinct and distributed brain regions, which is similar to the distribution of the DMN.^33^ Thus, impaired episodic memory in AD is not only due to the local damage of the hippocampus but also to impairment of networks (i.e., DMN) underlying memory procession. Anatomically, the lateral angular cortex connects to the MTL (i.e., hippocampus and parahippocampus) via the cingulum bundle and inferior longitudinal fasciculus.^34^, ^35^ Recently, an fMRI study investigated that the posterior angular cortex was functionally connected to the left anterior hippocampus, responding more strongly to items consistent with retrieval goals.^36^

We focused on the lateral angular cortex as a stimulation site because it is the portion of the hippocampal intrinsic fMRI connectivity networks that can be best targeted with rTMS because it is superficial. An rTMS‐fMRI study indicated that a multiple rTMS session protocol over the left angular cortex (based on connectivity in cortical‐hippocampal network) significantly improved memory performance, which was attributed to modulating synaptic plasticity.^19^ Another study in older adults also demonstrated that stimulation at the targeted location of the left angular cortex selectively modified neurobehavioral hallmarks of age‐related recollection impairment.^37^ These studies provided direct evidence for the potential utility of the left angular cortex as a simulation target to improve impaired memory function in patients with AD. Our cross‐sectional experiment proposed that the left angular area showing the weakest functional connectivity with the left hippocampus in AD could be the target site. According to this target, our longitudinal experiment indicated that rTMS induced a selective enhancement only in language and memory function. The exact patterns of functional rewiring of the brain connectome underlying cognitive enhancement will be described in detail below.

### 4.2 DMN subsystems divergently modulate the therapeutic effect of rTMS

The target‐based analysis in our study indicated that the brain regions in which alterations in connectivity were significantly associated with cognitive improvement after rTMS treatment were primarily distributed in the DMN. The DMN was the first functional network shown to be altered in AD.^33^ Recent evidence indicated that the DMN is a heterogeneous brain system comprising partially independent subsystems that serve dissociable cognitive processes.^31^, ^38^ By combining rs‐fMRI, graph analysis, and hierarchical clustering methods, Andrews‐Hanna et al. suggested that the DMN was segregated into three different subsystems: an MTL subsystem associated with memory retrieval, a dorsal medial prefrontal cortex (dMPFC) subsystem involved in social processing and a core subsystem related to self‐referential processes that acts as the hub linking the other subsystems together.^31^ In the current study, the MTL subsystem (ORBsupmed.L and ORBinf.B) and the dMPFC subsystem (SFG.L and MFG.R) displayed positive modulation, while the core subsystem (PCUN.B and ANG.B) presented negative modulation of the therapeutic effect of rTMS on memory and language cognition.

Previous rs‐fMRI studies indicated that the DMN subsystems in AD are not uniformly altered, and the MTL subsystem often exhibits hypoconnectivity, whereas the core subsystem exhibits hyperconnectivity.^39^, ^40^ Additionally, AD patients show a negative relationship between amplitudes of low‐frequency oscillations in the core subsystem of the DMN and episodic memory.^41^ The hyperconnectivity of the core subsystems may reflect compensatory activity as a result of Aβ deposition in the MTL subsystem early in the course of AD.^42^, ^43^ However, Jones et al. suggested that hyperconnectivity of the core subsystems is not compensatory but instead indicates the shift of burden to the core subsystem due to early MTL subsystem failures.^39^ Thus, we proposed that the negative correlation in the core subsystem between Δ connectivity and Δ cognition may be the protective mechanism of rTMS to relieve the overload and burden of hyperconnectivity, consistent with two recent TMS‐fMRI studies. One study found that rTMS‐induced hypoconnectivity between the posterior and anterior cingulate cortex (belonging to the core subsystem) was negatively correlated with improved memory function in AD.^44^ Another study using the intermittent theta‐burst TMS technique revealed that functional connectivity between the target and right precuneus cortex (belonging to the core subsystem) exhibited a dramatic decrease after stimulation, and this change was significantly related to enhancement in the language function.^45^ In addition, our study also demonstrated that the positive correlation between alterations of the MTL and dMPFC subsystems and improved cognition reflected significant beneficial rTMS effects in slowing cognitive dysfunction by modulating the functional activity of the target and its connectivity with the MTL and dMPFC subsystems. From a neurophysiological perspective, long‐term potentiation (LTP) is thought of a main correlation of cognition.^46^ TMS‐related studies have revealed decreased efficacy of LTP‐like neuroplastic mechanisms in AD from the early clinical stages.^47^, ^48^ The TMS‐electroencephalography study reported that high‐frequency rTMS could induce LTP‐like cortical plasticity within the DMN subsystems and induce enhanced TMS‐evoked β activity in AD.^12^ Therefore, although the DMN subsystems display distinct modulating patterns of rTMS therapy, the final outcome is unified to improve cognition by balancing the activity of DMN subsystems (deactivating the core subsystem and activating the MTL and dMPFC subsystems).

### 4.3 Intranetwork and internetwork functional connectivity modulate the therapeutic effect of rTMS

Functional changes related to the therapeutic effect of rTMS at the level of brain networks remain relatively poorly understood. In this part of the study, we are aimed to characterize the underlying mechanism of cognitive improvement after rTMS treatment from the perspective of whole‐brain functional networks. We found that the positive network related to improved cognition was characterized by internetwork connectivity between DMN and DAN, while the negative network was involved in intranetwork connectivity within the DMN. Specifically, the DMN, primarily supporting internally directed cognition, is engaged in the resting state, whereas the DAN, involved in external attention, typically shows increased activity during external attention‐demanding processing.^49^ These two networks exhibit inversely correlated activity across a range of different brain states.^50^ Recent AD investigations have reported that the DAN is functionally impaired to a greater extent than its ventral subsystem, suggesting that AD patients might have deficits in top‐down control processing rather than bottom‐up processing.^51^ Using dynamical causal modeling methodology, Chand et al. proposed that DAN regulates the functional activity between DMN and other networks to maintain normal cognitive processing, in accordance with the top‐down control role.^52^ Moreover, a longitudinal research provided evidence that the internetwork connections between DMN and DAN progressively decreased with disease progression from normal controls to late MCI.^53^ Thus, we speculate that the positive correlation between internetwork connectivity (between the DMN and DAN) and cognitive improvement reveals beneficial effects of rTMS stimulation by promoting neuroplasticity.

Among the large‐scale networks, the DMN was one of the first to be documented as a key network exhibiting dysfunction in AD, which is involved in internally directed and self‐referential cognition.^50^ Although our seed‐based analysis indicated that the DMN subsystems played divergent roles in modulating the rTMS therapeutic effect, the intranetwork connectivity within the DMN was primarily negatively correlated with improved cognition from the perspective of whole‐brain large‐scale networks. A study of rs‐fMRI in individuals with preclinical AD indicated that Aβ‐positive participants present significantly increased functional connectivity within the DMN.^54^ Based on the longitudinal Aβ‐PET study, Jack et al. Proposed that increased posterior DMN connectivity in individuals with low levels of Aβ who became amyloid‐positive at follow‐up.^55^ According to the cascading network mechanism described previously,^39^ we propose that the negative correlation between intranetwork connectivity within the DMN and improved cognition may be interpreted as a protective effect of rTMS treatment to resist the burden of AD‐related pathological markers, facilitating the processing of reallocating resources towards task‐positive functional networks.

### 4.4 Baseline functional connectivity predicts rTMS treatment response

It is critically important to be capable of predicting the possible response to rTMS stimulation using rs‐fMRI techniques. We utilized baseline connectivity as a predictive network according to the results of previous seed‐based and network‐based analyses. Our findings indicated that the connectivity patterns of DMN subsystems or large‐scale networks at baseline significantly predicted the therapeutic effect of rTMS. Among these, the connectivity patterns of large‐scale networks at baseline showed the best ability to predict the improvement of language function after rTMS treatment. Importantly, this good predictive capacity may further provide converging evidence of distinct modulation of DMN subsystems and large‐scale networks in response to rTMS intervention. A recent study focusing on rTMS‐induced connectivity within the DMN in AD patients illustrated a similar scenario.^44^ Furthermore, the baseline activity within the DMN was significantly negatively correlated with improved memory function. This means that subjects who presented better treatment response were those with relatively lower connectivity within the DMN at baseline.^44^ However, there is a lack of cross‐validation and permutation tests in this study, and the predictive ability of its model needs to be further investigated.^44^ Currently, it is difficult to select the appropriate intervention target before rTMS treatment to obtain the best therapeutic effect. Our findings might have far‐reaching significance in helping identify who preferentially responds to rTMS through baseline functional connectivity and choosing the stimulation target based on individualized prediction.

Our study is aimed to propose a new approach for revealing the underlying mechanism of rTMS therapy and predicting the rTMS therapeutic effect, but a few limitations need further investigations. First, the sample size of this study was relatively small, and no AD patients were recruited in the sham group. We will expand the sample size to validate these findings in our future studies. A multicenter longitudinal design is needed, and an individualized method for target selection will be formulated. Second, all participants in this study lacked cerebrospinal fluid examinations to identify potential pathological biomarkers. Third, all testing in this study was performed at the same site. Multiple‐site trials may yield improved and more precise data, and the potential mechanism may be more complex.

## 5 CONCLUSION

In this study, our findings reveal the efficacy of rTMS in the treatment of AD patients at the target of the left angular cortex and further explore altered functional connectivity induced by rTMS using longitudinally measured rs‐fMRI. Our findings suggest that the mechanism by which rTMS ameliorates cognitive deficits is by regulating the restrictive relationships between DMN subsystems and between the large‐scale functional networks associated with the DMN. Furthermore, dynamic regulation of the intra‐ and inter‐DMN at baseline may serve as a potential predictor of favorable rTMS response.

## AUTHOR CONTRIBUTIONS

FB designed this study. HFC and FB wrote the manuscript. HFC, XNS, ZYY, PFS, HHX, and RMQ analyzed the data. FB and HZ revised the manuscript. All authors read and approved the final manuscript.

## FUNDING INFORMATION

This work was supported partly by grants from the National Natural Science Foundation of China (No. 82071186; No. 82101539), Clinical Trials from the Affiliated Drum Tower Hospital, Medical School of Nanjing University (No. 2022‐LCYG‐MS‐05; No. 2021‐LCYJ‐PY‐30), National Key Research and Development Program of China (No. 2022YFA1105300), and Jiangsu Province Senior Health Project (No. LKZ2023014).

## CONFLICT OF INTEREST STATEMENT

All authors have no conflicts of interest.

## Supporting information


Appendix S1

## Data Availability

The data used to support the findings of this study are available from the corresponding author upon request.
