# Supplementary material for: Multi‐networks connectivity at baseline predicts the clinical efficacy of left angular gyrus‐navigated rTMS in the spectrum of Alzheimer's disease: A sham‐controlled study
Source: CNS Neurosci Ther. 2023 Mar 21;29(8):2267–80. doi: 10.1111/cns.14177 (PMC10352882; doi:10.1111/cns.14177)
Supplement: Supplementary file 1 — Appendix S1 [file CNS-29-2267-s001.doc]

**Supplementary Materials**

***Participants***

The possible or probable AD was diagnosed based on the National Institute of Neurological and Communicative Disorders and Stroke and the AD and Related Disorders Association (NINCDS-ADRDA) and the Diagnostic and Statistical Manual of Mental Disorders IV criteria (DSM-IV) guidelines. Other key inclusion criteria included the scores for the Mini-Mental State Examination (MMSE) < 24 and clinical dementia rating (CDR) score 1 or 2. The aMCI patients included in this study were diagnosed according to the recommendations of Petersen and described as follows: (1) memory complaint confirmed by the subject and/or an informant; (2) objective cognitive performance documented by an auditory verbal learning test-delayed recall (AVLT-DR) scores below or equal to 1.5 SD of education- and age-adjusted norms; (3) CDR score = 0.5; (4) MMSE scores ≥ 24; and (5) not sufficient to dementia according to NINCDS-ADRDA and DSM-IV. More detailed information about the criteria of aMCI has been described in our previous study. The HC subjects were required to have MMSE scores ≥ 26 and CDR score of 0. In the longitudinal experiment, eighteen participants (13 aMCI and 5 AD patients) in rTMS group and six participants (6 aMCI patients) in sham group were included in the final analysis. To avoid the confusion, we list the MMSE scores of aMCI and AD patients separately in the longitudinal experiment (shown in the Supplementary Table 2).

***Neuropsychological measurement***

General cognitive functioning was evaluated by MMSE, the Beijing version of the Montreal Cognitive Assessment (MoCA-BJ) and CDR. The multiple cognition domains including episodic memory, executive function, language function, information processing speed and visuospatial function were also evaluated. Memory function was calculated as the mean of the Z-scores from the Visual Reproduction-delayed recall (VR-DR), AVLT-DR and AVLT-recognition (AVLT-R). Executive function is a compound score of the average Z-scores of the Digit Span Test-backward (DST-backward), Trail Making Test-B (TMT-B) and Stroop Color and Word Tests C (Stroop C). The language function consisted of the Category Verbal Fluency (CVF) and Boston Naming Test (BNT). Information processing speed was calculated as the average Z-scores of TMT-A, Stroop A and Stroop B. Visuospatial function is a compound score that includes the mean of the Z-scores of the Clock Drawing Test (CDT) and Visual Reproduction-copy (VR-C).

***MRI scanning***

All of the subjects were examined on a 3.0T MRI scanner (Philips Medical Systems). The protocol included the high-resolution T1-weighted imaging [echo time = 4.6 ms, repetition time = 9.8 ms, ﬂip angle = 8°, field of view = 250×250 mm2, acquisition matrix = 256×256, number of slices = 192, thickness = 1.0 mm] and a gradient-recalled echo planar imaging sequence (repetition time = 2000 ms, ﬂip angle = 90°, echo time = 30 ms, number of slices = 35, acquisition matrix = 64 × 64, field of view = 240 × 240 mm2 , thickness = 4 mm). During the rs-fMRI scans, all subjects were instructed to keep their eyes closed, relax and move as little as possible, and to not fall asleep.

***Validation***

To valid the stability of our result, one-way ANCOVA analysis was performed again with gender, age, years of education and head motion (e.g., FD-Jenkinson) as covariates in the cross-sectional experiment. We found that only one cluster exhibited group differences (*p* < 0.05, cluster size = 540 mm3) (Supplementary Figure 4). The centre of this cluster (MNI coordinate: -39, -63, 27) is also located in the angular cortex belonging to the parietal region.

**Supplementary Table 2**

**MMSE scores of aMCI and AD patients separately in the longitudinal experiment**

| **Items** | **rTMS group (aMCI = 13, AD = 5)** | | | | **Sham group (aMCI = 6)** | | | |
| --- | --- | --- | --- | --- | --- | --- | --- | --- |
|  | **Pre** | | **Post** | | **Pre** | | **Post** | |
|  | **aMCI** | **AD** | **aMCI** | **AD** | **aMCI** | **AD** | **aMCI** | **AD** |
| **MMSE** | 27.62±1.66 | 18.40±4.39 | 28.00±2.08 | 21.00±4.90 | 29.33±1.03 | -- | 28.83±1.17 | -- |

Values are presented as the mean ± standard deviation (SD).

Abbreviations: AD, Alzheimer’s disease; aMCI, amnestic mild cognitive impairment; rTMS, repetitive transcranial magnetic stimulation; MMSE, mini mental state examination.

**Supplementary Table 3**

**Supplementary Figure 1**


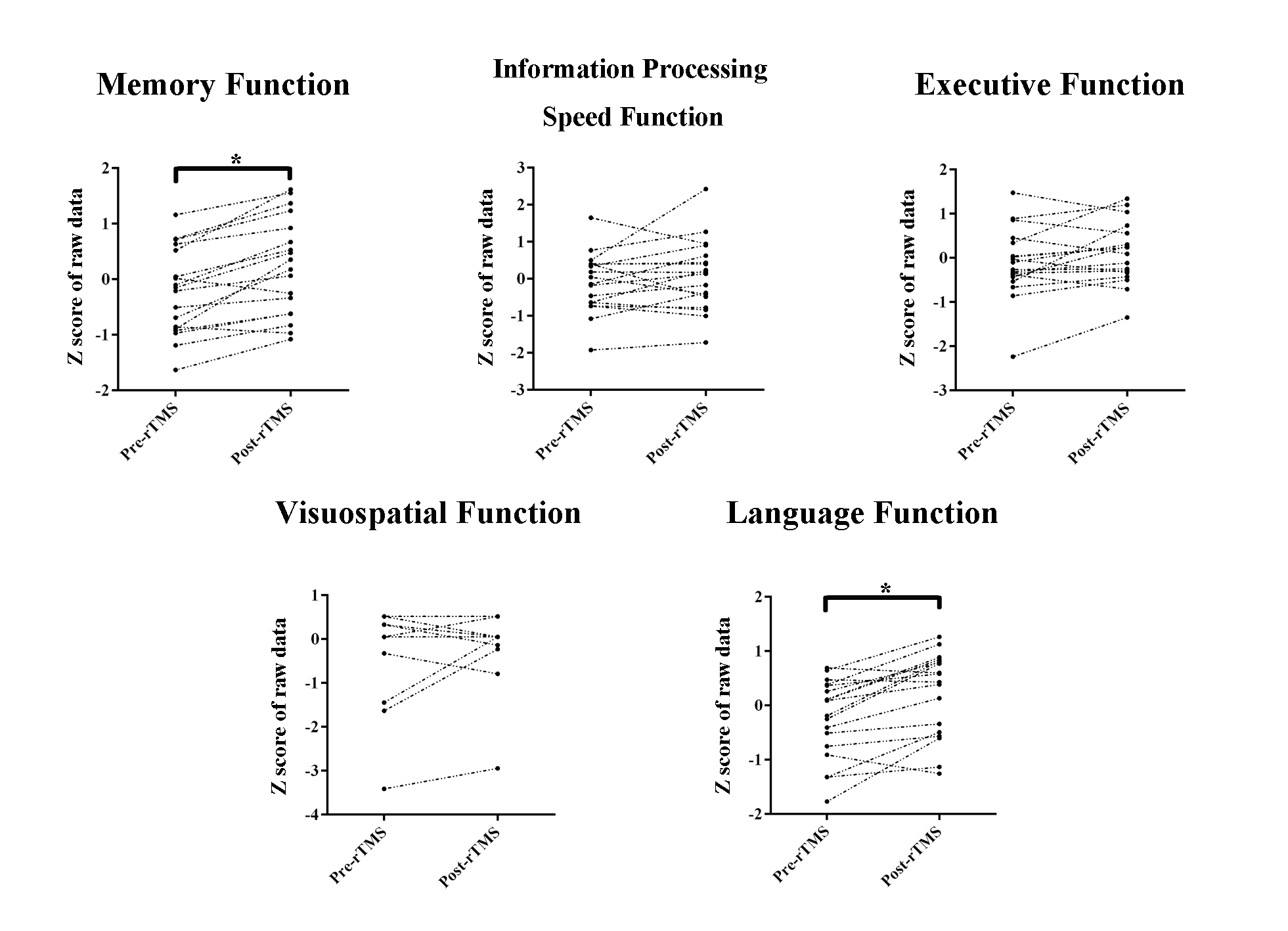


Cognitive performance before and after rTMS treatment are summarized. Statistically significant improvements were seen when the pre- and post-stimulation cognitive performance were compared using a paired *t*-test. The stimulation target induced improvement in the MoCA-BJ (*t* = 4.64, *p* < 0.001), the memory function (*t* = 5.36, *p* < 0.001), and the language function (*t* = 4.74, *p* < 0.001).


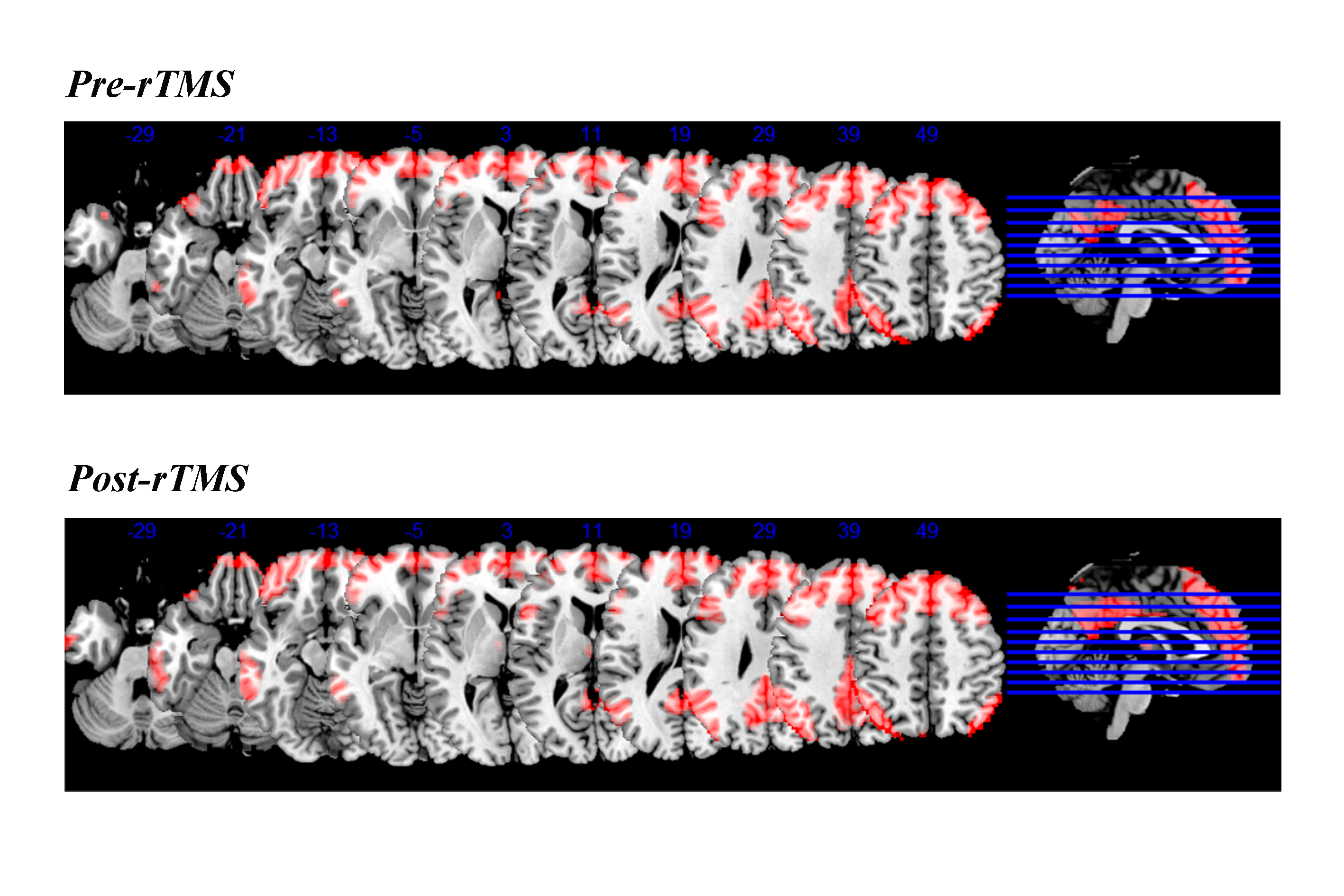
**Supplementary Figure 2**

Functional networks based on the stimulation target before and after rTMS treatment.

**Supplementary Figure 3**

**
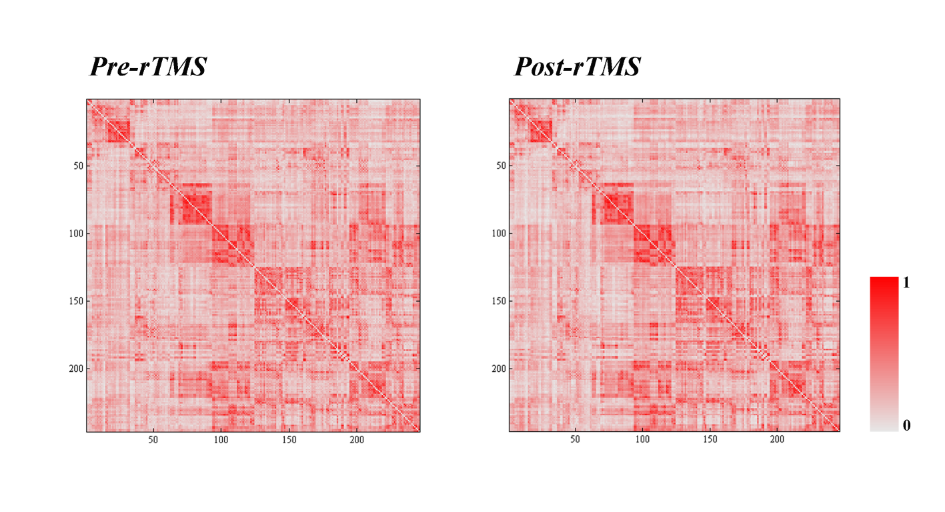
**

Whole brain functional networks before and after rTMS treatment.

**Supplementary Figure 4**


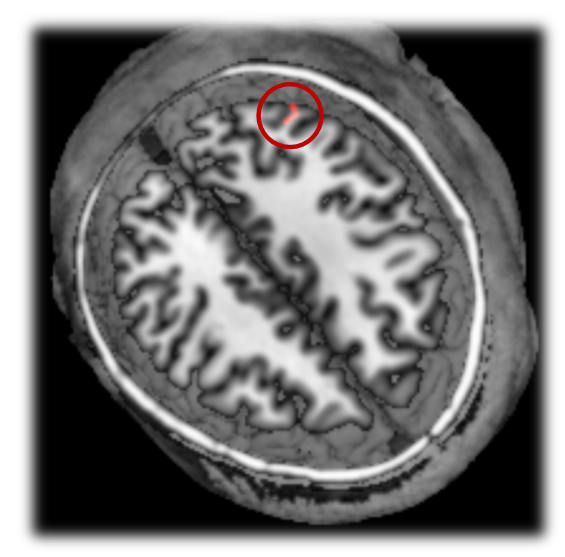


Significantly different cluster after gender, age, years of education and head motion as covariates (peak MNI coordinate: -39, -63, 27; *p* < 0.05, cluster size = 540 mm^3^).
